# Exposure to trauma-relevant pictures is associated with tachycardia in victims who had experienced an intense peritraumatic defensive response: the tonic immobility

**DOI:** 10.3389/fpsyg.2014.01514

**Published:** 2014-12-23

**Authors:** Rita de Cassia S. Alves, Liana C. L. Portugal, Orlando Fernandes Jr, Izabela Mocaiber, Gabriela G. L. Souza, Isabel de Paula A. David, Eliane Volchan, Leticia de Oliveira, Mirtes G. Pereira

**Affiliations:** ^1^Laboratory of Neurophysiology of Behavior, Department of Physiology and Pharmacology, Biomedical Institute, Federal Fluminense UniversityNiterói, Brazil; ^2^Laboratory of Psychophysiology, Department of Biological Sciences, Federal University of Ouro PretoOuro Preto, Brazil; ^3^Laboratory of Neurobiology II, Institute of Biophysics Carlos Chagas Filho, Federal University of Rio de JaneiroRio de Janeiro, Brazil

**Keywords:** emotion, defensive responses, tonic immobility, heart rate, trauma-relevant pictures, individual difference, stimuli relevance

## Abstract

Tonic immobility is an involuntary, last-ditch defensive reaction characterized by physical inactivity in a context of inescapable threat that has been described in many species, including humans. The occurrence of this defensive response is a predictor of the severity of psychiatric disorders and may be considered as an index of an intense reaction to a traumatic event. Here, we investigated whether the retrospective reports of peritraumatic tonic immobility reaction in participants exposed to a traumatic event would modify their cardiac responses to pictures related to their trauma. Using a questionnaire of life-threating events, we selected students who experienced violent crime as their most intense trauma and students who had never experienced a violent crime trauma, but experienced other traumatic events. All participants completed a questionnaire that estimated the intensity of tonic immobility during their most intense trauma. Electrocardiographic recordings were collected during exposure to pictures. Participants viewed emotional pictures (human attack with guns) and neutral pictures. These emotional stimuli were selected to be trauma-relevant to the violent crime group and non trauma-relevant to the no violent crime trauma group. Violent crime group showed a positive correlation between heart rate changes after viewing trauma-related pictures and tonic immobility scores. We observed that low tonic immobility scores were associated with bradycardia and high scores with tachycardia in response to trauma-relevant pictures. For the no violent crime group, no significant correlation was detected. These results suggest that the relevance of the stimuli and the magnitude of the defensive response during a previous trauma event were important factors triggering more intense defensive responses.

## INTRODUCTION

Tonic immobility is an innate behavioral response characterized by temporary, profound physical inactivity, analgesia, and relative unresponsiveness to external stimulation that occurs in response to inescapable threats (Ratner, 1967; Klemm, 1971; Gallup and Rager, 1996). Ratner (1967) proposed that defensive responses are widely conserved in the animal kingdom and, according to the defensive distance model, are organized in a sequence of defensive events, starting with freezing and progressing to flight, fight, and tonic immobility. Tonic immobility has been considered the ultimate defensive reflex against capture by a predator (Ratner, 1967; Marx et al., 2008). This response may increase the chances of survival by preventing or at least reducing, the likelihood of new attacks because the chances of a predator attack decrease with the cessation of prey-specific responses, such as struggling and vocalizations (Gallup, 1974; Marks, 1987).

The tonic immobility response has been documented in animals for more than three centuries (Maser and Gallup, 1977). In humans, studies have demonstrated that tonic immobility also occurs, especially during a life-threatening event (Suarez and Gallup, 1979; Galliano and Noble, 1993; Heidt et al., 2005; Fusé et al., 2007; Bados et al., 2008; Bovin et al., 2008; Abrams et al., 2009; Humphreys et al., 2010; Portugal et al., 2012). Recently, studies found that tonic immobility was a predictor of the severity of posttraumatic stress disorder (PTSD) symptoms and of poor response to pharmacological treatment in PTSD patients who had been exposed to urban violence (Fiszman et al., 2008, Rocha-Rego et al., 2009; Lima et al., 2010). Moreover, tonic immobility was significantly associated with PTSD symptoms in a non-clinical sample (Portugal et al., 2012). Taken together, these studies suggest that the occurrence of this defensive reaction is not uncommon in humans and its occurrence might reflect how intense the traumatic event is perceived by the individual.

To the best of our knowledge, Volchan et al. (2011) conducted the first study that linked verbal reports of tonic immobility with objective measures of body immobility. The authors evaluated trauma-exposed participants with and without PTSD and used posturography and electrocardiography measures to record their reactions to an audio-play script of autobiographical trauma. Reports of script-induced immobility were associated with reduced area of body sway and were correlated with an accelerated heart rate. Re-experiencing tonic immobility in the laboratory was more evident in participants with PTSD. Heart acceleration was consistent with descriptions of heart rate modulation during tonic immobility induction in other species (Ratner, 1967; Carli, 1974; Valance et al., 2008). This evidence supports the idea that tonic immobility is retained in humans as a defensive strategy for responding to trauma cues. An important open question is if the occurrence of tonic immobility during a traumatic event would modify physiological responses (e.g., heart rate response), when participants from a non-clinical sample are exposed to trauma-relevant pictures.

Studies have consistently described heart rate deceleration that occurs when healthy participants view unpleasant pictures in the laboratory (Bradley et al., 2001; Azevedo et al., 2005; Facchinetti et al., 2006; Löw et al., 2008; Mocaiber et al., 2011). This cardiac deceleration response is hypothesized to be part of a freezing-like defensive response (Cuthbert et al., 1996; Bradley et al., 2001). By employing posturography and electrocardiography, “freezing” during the viewing of unpleasant pictures was characterized by reduced body sway and heart rate deceleration (Azevedo et al., 2005; Facchinetti et al., 2006). According to a defense cascade model for humans under picture viewing in laboratory conditions, which was proposed by Lang et al. (1997), a transition from defensive freezing to a more overt reaction can occur when the threat increases, even with a switch from bradycardia (cardiac deceleration compared to the baseline) to tachycardia (cardiac acceleration compared to the baseline) (Lang, 1995; Lang et al., 1997, 2000). In addition, individual differences in threat perception feed into this transition. For instance, phobic individuals experience cardiac acceleration when viewing pictures of their phobic object (Klorman et al., 1977; Hamm et al., 1997; Wendt et al., 2008; Sebastiani et al., 2014). Individual differences may be a key factor in generating different physiological responses to emotional stimuli.

In the present study, we investigated individual differences in the heart rate responses that occurred in a defensive context. Specifically, we studied whether retrospective reports of the magnitude of the tonic immobility reaction during exposure to a traumatic event would modify the heart rate response to trauma-relevant pictures. We hypothesized that individuals who manifested a high level of tonic immobility during a traumatic event would experience a different defensive response to trauma-relevant pictures. Because tonic immobility is experienced only in extreme threat situations, these individuals may become more responsive to trauma-relevant stimuli, triggering more intense defensive responses. We recruited healthy college students who had been victims of violent crime and, as controls, students who had never experienced such trauma, but experienced other traumas. We chose a non-clinical sample, because this sample reduces the high prevalence biases, such as comorbidities, medication, and high levels of functional impairment, that are commonly observed in clinical samples.

## MATERIALS AND METHODS

### PARTICIPANTS

Participants were undergraduate students at Fluminense Federal University. They were selected using a purposive sampling technique, which targets a particular group of people. In the present study, selection was based on the types of life-threatening trauma to which they had been exposed. In the pre-experimental phase, 73 participants (52 females) completed a traumatic events checklist and were informed that they might be invited to participate in the next step. We invited all participants who had either experienced a “violent crime” (see details below in psychometric measures) as their most intense lifetime trauma or those who had never experienced a traumatic “violent crime” event, but had other traumas. According to these criteria, 37 participants were selected.

All participants had normal or corrected vision. Participants were naive with regard to the purpose of the experiment. The local ethics committee approved the experimental protocol, and each participant gave written consent prior to the study. Participants were informed that they could withdraw from the experiment at any time.

### APPARATUS AND STIMULI

The participants were tested in a sound-attenuated room under dim ambient light. Stimulus timing and presentation were programmed using the E-Prime® software (Psychology Software Tools Inc., Pittsburgh, PA, USA). During the experiment, the participant’s head was positioned on a head- and chin-rest that was 57 cm from the screen.

Sixty-four photos (32 human attack and 32 human neutral) were used. The emotional stimuli were photographs of people using weapons, and the neutral stimuli were photographs of people in everyday situations. The pictures were obtained from the Internet, purchased from Getty Images® (http://www.gettyimages.com) or photographed by the authors, with the exception of one picture that was obtained from the International Affective Picture System (IAPS; Lang et al., 2005).

The neutral and human attack stimuli were matched in terms of picture composition (e.g., number of faces, gender, body parts, people, and ethnicity) to ensure that emotional content was the only attribute that differed between the two image categories. Each emotional stimulus was paired with a neutral stimulus. Human attack and neutral pictures were also matched by complexity and perceptual properties (brightness, contrast, and spatial frequency). This procedure was conducted because a previous study had shown that picture complexity (clear figure-ground pictures compared with complex scenes that depicted multiple objects) rather than emotionality was responsible for some of the differences observed in the recorded neural responses to neutral and emotional pictures (Bradley et al., 2007). We attempted to minimize this confounding factor by selecting only emotional and neutral stimuli with approximately the same level of complexity, i.e., they were all clear figure-ground pictures with the same perceptual properties. To assess the adequacy of this a priori selection, we followed the procedures of Bradley et al. (2007) and asked an independent sample of 58 students (42 female) to rate picture complexity on a scale of 1–9 (1 = clear figure-ground, 9 = complex scenes). The results corroborated our *a priori* selection of pictures. The mean complexity of the human attack (with guns) and neutral stimuli did not differ (*p* = 0.33, see **Table 1**). The physical properties of the human attack and neutral pictures also did not differ with regard to brightness (*p* = 0.69), contrast (*p* = 0.17), and spatial frequency (*p* = 0.41; see **Table 1**).

**Table 1:** Means and SD of valence, arousal, complexity, brightness, contrast, and spatial frequency values for neutral and human attack stimuli.

	Human attack (with guns) (SD)	Neutral (SD)
Valence	2.28 (1.22)	5.29 (0.99)
Arousal	6.18 (2.19)	3.68 (2.00)
Complexity	2.88 (0.81)	3.09 (0.88)
Brightness	84.16 (30.27)	81.21 (28.67)
Contrast	19.87 (8.08)	22.74 (8.62)
Spatial frequency	0.97 (0.08)	0.99 (0.13)

Following the protocol developed by Lang et al. (1997), the valence and arousal value of the pictures were rated on a scale of 1–9 by a separate group of 134 participants (104 female, 21.5 years ± 3.36) using the paper-and-pencil version of the Self-Assessment Manikin (Bradley and Lang, 1994). The mean values of valence and arousal for each picture category are shown in **Table 1**.

Human attack pictures are considered as trauma-relevant stimuli for the participants who had experienced a “violent crime” as their most intense lifetime trauma and non trauma-relevant for those who had never experienced a traumatic “violent crime” event.

### DESIGN AND PROCEDURE

After providing written informed consent, participants were seated at a table in a sound-attenuated, temperature-controlled (22–24°C) room. ECG electrodes were placed on the chest at lead II. Task instructions were given, and the experimental session was initiated.

The experimental session consisted of two blocks, each with 16 human attack stimuli (e.g., person with a gun) and 16 paired neutral stimuli (e.g., person with an object). The order of the blocks was randomized, and the presentation of neutral and human attack (with guns) pictures within the experiment was pseudo-randomized among participants. Each trial began with a fixation cross that was presented for 6–8 s, which was followed by a picture presented for 6 s. Participants were instructed to observe the picture during its presentation.

Each participant was first familiarized with the paradigm before performing the experimental block of passive viewing. An additional set of 13 pictures from the IAPS (eight of animal attacks, three of nature, and two of mushrooms) were used to familiarize participants with the experimental setup.

### PHYSIOLOGICAL MEASUREMENTS

Two PC-compatible computers were used. One computer was used to control electrocardiographic data acquisition (Acknowledge-BIOPAC Systems Inc.), and the other computer was used to present the pictures (E-Prime® software-Psychology Software Tools Inc., Pittsburgh, PA, USA).

Electrocardiographic recordings were collected at a band filter of 0.05–60 Hz and a sampling frequency of 500 Hz through an electrocardiograph ECG100C module coupled to the MP150 system (BIOPAC Systems Inc., Santa Barbara, CA, USA). An off-line peak detection algorithm (derivative plus threshold) was used to estimate R-wave fiducial points, and afterwards, the series was screened by hand and corrected for artifacts. A fractional cycle counts algorithm (Dinh et al., 1999) was applied to calculate heart rate values at 0.5-s intervals during the 6-s picture presentation (12 values). Heart rate modulation was obtained by subtracting each heart rate value from that measured during the 1-s period before picture presentation (baseline period; Bradley et al., 2001). The mean of 12 values was then calculated to produce one value per picture.

Data processing followed the recommendations of the Task Force of the European Society of Cardiology and the North American Society of Pacing and Electrophysiology (1996). We used the Matlab software KARDIA (MathWorks Inc., Natick, MA, USA) to analyze the cardiac parameters (Perakakis et al., 2010).

### PSYCHOMETRIC MEASURES

We used the Trauma History Questionnaire (THQ; Green, 1996; translated and adapted to Portuguese by Fiszman et al., 2005) to evaluate the frequency and types of life-threatening trauma to which the participants had been exposed. The THQ is a list of 23 items that fall within a range of potentially traumatic events in three domains: crime-related events (e.g., robbery and mugging), general disaster and trauma (e.g., injury, disaster, and witnessing death), and unwanted physical and sexual experiences. The questionnaire also contains an open-ended question that allows participants to specify other extraordinarily stressful situations or events that they have experienced. The instructions were to respond whether the participant had experienced each of the event types during his/her lifetime. The items from the THQ that are considered to be “violent crimes” were selected according to the trauma categorization of Luz et al. (2011) and included physical/violent assault, crime/violence victims, community/workplace/urban interpersonal violence, robbery, shooting, or aggression. Because we employed pictures of a gun attack as trauma-relevant stimuli, “sexual assault,” “violence in war situations,” “domestic violence,” and “child abuse” were not included in the criteria to select participants exposed to “violent crimes.”

We also used a scale that was an adaptation of the four “motor” items (TIS-4, Lima et al., 2010; Volchan et al., 2011) from the Tonic Immobility Scale - Child form (TIS-C; Forsyth et al., 2000). Each question was evaluated on a likert scale that ranged from 0 to 6. The four “motor” items used were: (1) the degree to which you froze or felt paralyzed during the event (0 = not at all frozen or paralyzed, 6 = completely frozen or paralyzed), (2) the degree to which you were unable to move even though you were not restrained during the event (0 = could move freely, 6 = could not move at all), (3) the degree to which you were unable to call out or scream during the event even though you wanted to call out (0 = felt able to scream, 6 = wanted to scream but felt unable to), and (4) the extent to which you felt unable to escape during the event even though you wanted to escape (0 = felt able to escape, 6 = wanted to escape but remained fixed). We calculated the means and SDs of the scores on the four “motor” items (minimum = 0, maximum = 24) of the TIS-4. We were particularly interested in investigating how the magnitude of a peritraumatic defensive response, tonic immobility, would modulate the cardiac response to trauma-relevant pictures in individuals who had experienced violent crimes that they regarded as the most intense trauma of their lives, and in individuals who had not experienced traumatic violent crimes, but experienced other traumas.

### DATA ANALYSIS

For each participant, we averaged the heart rate modulation across all pictures for each category. We conducted Spearman correlation analyzes to investigate the association between the tonic immobility scores and heart rate changes that occurred while viewing human attack (with guns) and neutral pictures. We conducted these analyzes separately for the samples with and without violent crime as traumatic event. The alpha level for statistical significance was set at α = 0.05.

## RESULTS

### SAMPLE CHARACTERISTICS

Five participants were excluded because of technical problems in data acquisition, and one subject was excluded because of cardiac arrhythmias. Two participants were excluded for showing a mean of heart rate in response to pictures higher or lower than the average by ±3 SD. The final sample consisted of 29 students, of which 14 (mean age: 20.7 ± 2.7 years old, 10 women) had experienced violent crime as their most intense lifetime trauma, and 15 (mean age: 19.4 ± 1.0 years ols., 11 women) had experienced other traumas but not a traumatic violent crime event. For the latter participants, the most traumatic life events reported in the THQ included death or loss of someone close to the respondent (*n* = 6), a medical event (*n* = 3), sexual abuse (*n* = 2), a vehicle accident (*n* = 2), injury (*n* = 1), and other (*n* = 1).

The mean score of retrospective reports on the TIS-4 was 11.6 (SD = 6.6, min = 0, max = 24). The mean tonic immobility scores for the violent crime group (*M* = 12.3, SD = 7.1, range = 0–21) and no violent crime group (*M* = 11.4, SD = 6.7, range = 0–20) did not differ, *t*(27) = 0.32, *p* = 0.75. Additionally, ratings of trauma intensity obtained via the THQ scale indicated that the violent crime group (*M* = 4.14, SD = 1.03) and no violent crime group (*M* = 4.29, SD = 0.99) did not differ, *t*(26) = -0.38, *p* = 0.71.

### HEART RATE

**FIGURE 1:**
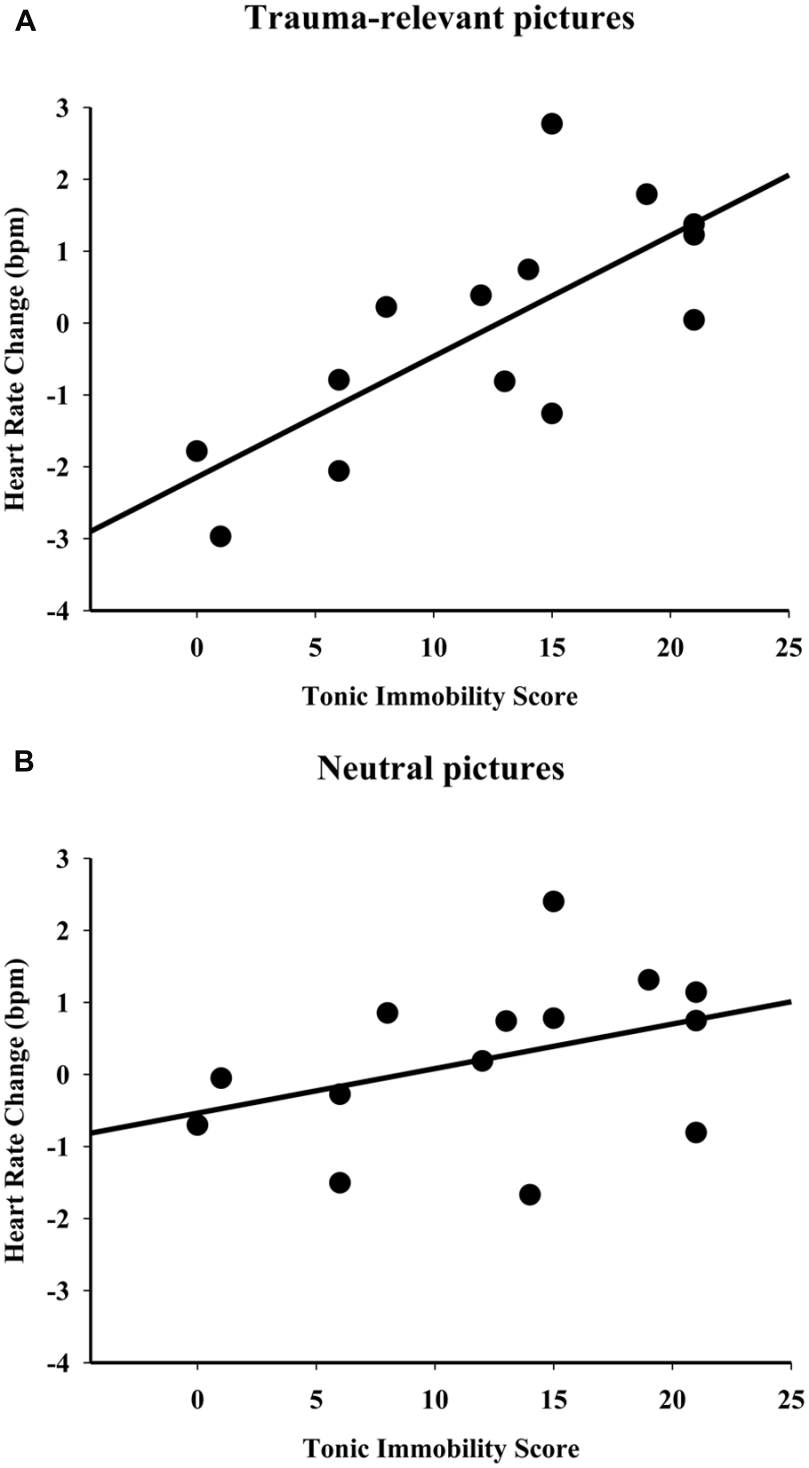
**Correlations in participants who had experienced violent crime as their most intense lifetime trauma. (A)** Positive correlation between the tonic immobility scores and heart rate changes that occurred while viewing trauma-relevant pictures (human attack pictures, *r* = 0.70, *p* < 0.05). **(B)** No significant correlation was observed between the tonic immobility scores and heart rate changes that occurred while viewing neutral pictures. The values of the tonic immobility scores represent the means per subject of the four “motor” items on the Tonic Immobility Scale (TIS-4).

In the group of participants who had experienced violent crime as their most intense trauma, a positive correlation was observed between the TIS-4 scores and heart rate changes that occurred while viewing trauma-relevant pictures (human attack pictures) (ρ = 0.70, *p* = 0.005, **Figure 1A**). Interestingly, low scores on the TIS were associated with bradycardia, and high scores on the TIS were associated with tachycardia. No significant correlation was observed between the TIS-4 scores and heart rate changes that occurred while viewing neutral pictures (ρ = 0.43, *p* = 0.13, **Figure 1B**).

**FIGURE 2:**
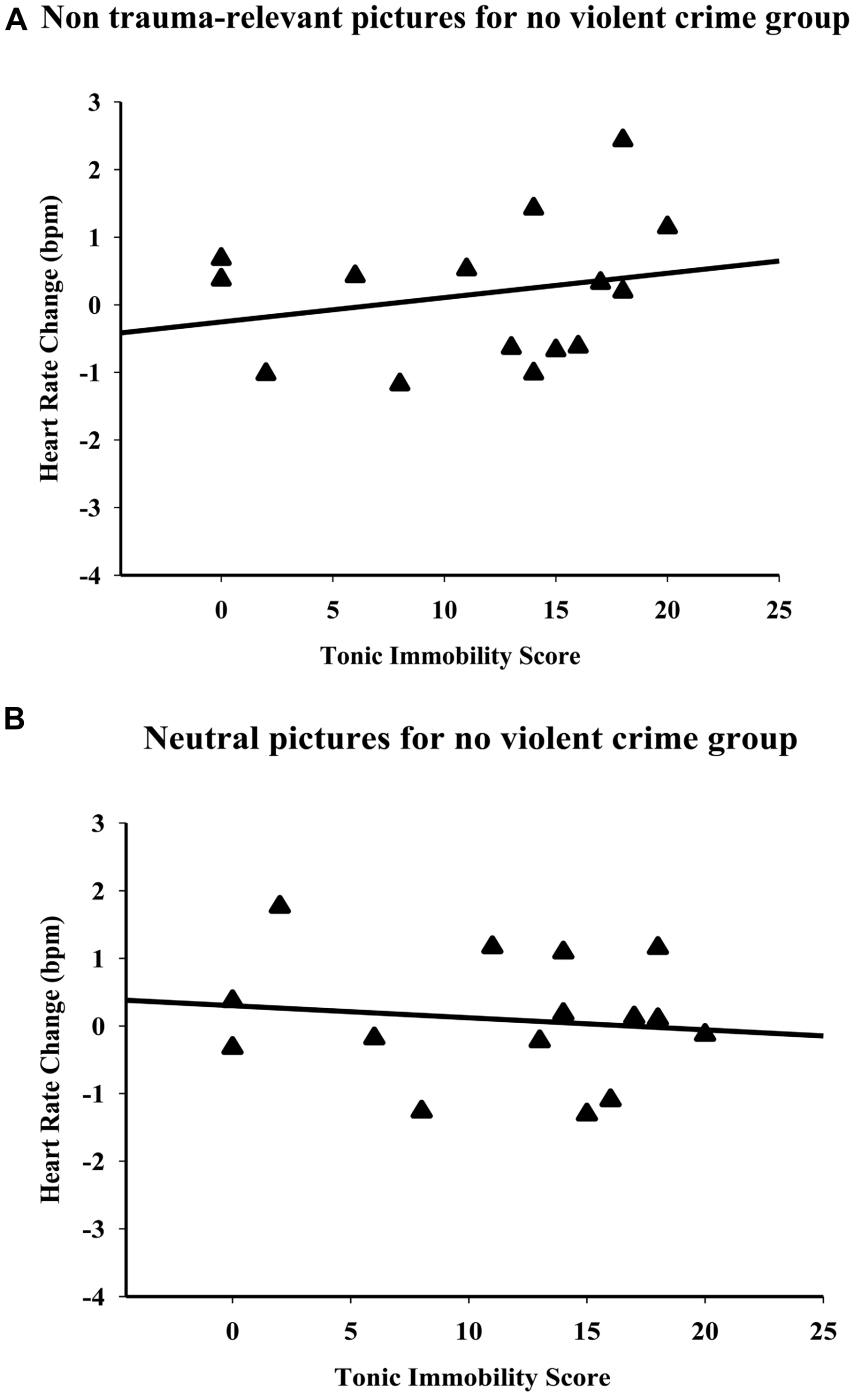
**Correlations in participants who had never experienced violent crime, but had other traumas.** No significant correlation was detected between the tonic immobility scores and heart rate changes that occurred while viewing non trauma-relevant **(A)** and neutral pictures **(B)**. The values of the tonic immobility scores represent the means per subject of the four “motor” items on the TIS-4.

In the group of participants who had other traumas but never undergone a traumatic violent crime, no significant correlation was observed between heart rate changes that occurred while viewing human attack pictures (non trauma-relevant pictures) and the TIS (ρ = 0.22; *p* = 0.43, **Figure 2A**). No significant correlation was observed while participants were exposed to neutral pictures either (ρ = -0.08; *p* = 0.86, **Figure 2B**).

## DISCUSSION

Our findings indicate that the level of tonic immobility, an intense peritraumatic response, retrospectively reported is associated with the type of cardiac response presented by participants when exposed to trauma-relevant pictures. We used a non-clinical sample of victims of violent crime that the victims regarded as their most intense lifetime trauma and a second group of participants who had experienced other traumas but never experienced a traumatic violent crime event. We found significant positive correlations between TIS-4 scores and heart rate changes while viewing human attack pictures for the group presented with trauma-relevant pictures. Low tonic immobility scores were associated with bradycardia, and high tonic immobility scores were associated with tachycardia. In contrast, for the group that the emotional stimuli were non trauma-relevant (participants who had never experienced a violent crime trauma event) no significant correlation was observed between heart rate changes and TIS-4 scores while viewing human attack or neutral pictures.

Interestingly, the tonic immobility scores and the intensity of trauma did not differ between groups. Thus, it seems that it is not a difference of average intensity of trauma that is responsible for the presence of a correlation between TIS-4 scores and heart rate response only in the violent crime group. This result corroborates the idea that the relevance of the emotional stimuli was a key factor to reveal this association. In this same vein, stimulus type has been shown relevant for individuals when studying phobia, which showed cardiac acceleration when viewing pictures of their phobic object (Klorman et al., 1977; Hamm et al., 1997; Wendt et al., 2008; Sebastiani et al., 2014). Furthermore, samples of traumatized PTSD patients have also been reported to experience marked heart rate acceleration in response to pictures with trauma cues (Elsesser et al., 2004; Ehlers et al., 2010). However, other studies have shown that behavioral interference is also dependent on stimuli relevance (MacLeod et al., 1986; Mocaiber et al., 2009; Purkis et al., 2011; Fernandes et al., 2013; Stout et al., 2013). For example, a recent study by Purkis et al. (2011) demonstrated that the interference produced by emotional pictures was dependent on the relevance of these stimuli for each individual (for review, see also Oliveira et al., 2013).

Anxiety studies have also shown that anxious participants exhibit greater interference in response to threat-related stimuli and that difficulty in filtering threat-related distracters was exaggerated among anxious individuals (e.g., MacLeod et al., 1986; Mocaiber et al., 2009; Stout et al., 2013). A meta-analysis conducted by Bar-Haim et al. (2007) identified several studies that have shown a bias in processing stimuli by individuals with PTSD that is related to their trauma (a threat-related bias; McNally et al., 1990; Bryant and Harvey, 1995; Buckley et al., 2000). Studies using script driven imagery paradigms find intense reactions to trauma-related stimuli in PTSD subjects (see Lobo et al., 2011 for a review). A recent finding showed that the emotional modulation effect produced by threat stimuli was influenced by the number and types of violent crime that had previously been experienced by the participant (Fernandes et al., 2013). The authors suggested that the impact of threat stimuli on participants’ behavior is dependent on the extent to which the participant considers the stimuli relevant (Fernandes et al., 2013).

There were some limitations in the present study. First, although a relationship between tonic immobility scores and heart rate change emerged, this might be considered as a preliminary finding due to our relatively modest samples. Due to concerns about false positives, further work with larger samples could verify the current findings. Second, the heterogeneity of traumas in the no violent crime trauma group might have increased the variability of responses when participants faced human attack pictures. Third, even though passive viewing is a frequently used task in emotion literature, we did not control attentional vigilance during the task.

In summary, our results suggest that the magnitude of the occurrence of an intense peritraumatic response (i.e., tonic immobility response), during a previous traumatic event affects subsequent emotional responses to trauma-relevant stimuli. Tonic immobility is triggered by perception of very intense life risk during a traumatic event. Cumulative clinical evidence has linked this defensive reaction to the severity of the most disruptive sequela of trauma exposure, PTSD. The occurrence of high levels of tonic immobility in victims is an evidence that the participant perceived the context as extremely threatening. Therefore, experiencing high levels of tonic immobility in the past certainly points to an escalation in the defensive cascade and to re-experiencing a context of great danger when facing trauma-related cues in the future.

## AUTHOR CONTRIBUTIONS

Mirtes G. Pereira, Eliane Volchan, Rita de Cassia S. Alves, and Leticia de Oliveira conceptualized the study. All authors designed the study Rita de Cassia S. Alves collected data and analyzed data. All authors contributed to data interpretation. Mirtes G. Pereira, Leticia de Oliveira, and Rita de Cassia S. Alves and Liana C. L. Portugal wrote the paper. Mirtes G. Pereira and Orlando Fernandes Jr created the figures and table. Rita de Cassia S. Alves inserted references. Mirtes G. Pereira and Leticia de Oliveira supervised the study. All authors contributed extensively to revising the paper.

## Conflict of Interest Statement

The authors declare that the research was conducted in the absence of any commercial or financial relationships that could be construed as a potential conflict of interest.
